# Allelopathic Effects of *Bidens pilosa* L. var. *radiata* Sch. Bip. on the Tuber Sprouting and Seedling Growth of *Cyperus rotundus* L.

**DOI:** 10.3390/plants9060742

**Published:** 2020-06-12

**Authors:** Ming-Tung Hsueh, Chihhao Fan, Wen-Lian Chang

**Affiliations:** 1 Department of Bioenvironmental Systems Engineering, National Taiwan University, Taipei 106, Taiwan; d02622002@ntu.edu.tw (M.-T.H.); wenlian@ntu.edu.tw (W.-L.C.); 2 Taitung District Agricultural Research and Extension Station, Taitung 950, Taiwan

**Keywords:** *Bidens pilosa* L. var. *radiata* Sch. Bip. (*BPr*), *Cyperus rotundus* L. (*CR*), allelopathy, weed control, interspecies competition, density-dependent phytotoxicity

## Abstract

*Bidens pilosa* L. var. *radiata* Sch. Bip. (*BPr*) had been found capable of excluding *Cyperus rotundus* L. (*CR*) from its vegetation in fallow fields. Both allelopathy and competition of *BPr* were able to limit the growth of *CR*, but this has not been extensively investigated. To verify the two effects of *BPr* on *CR* management, density-dependent experiments and interspecies competitions with the application of activated carbon were conducted. The effects of *BPr* soil and its residues on the reproduction of *CR* were also evaluated. The results showed that the residues of *BPr* reduced the growth (54–61% of control) and tuber number (58–71% of control) of *CR* in the 3 plants pot^−1^ treatment but not in higher density treatments. In the interspecies competition, *BPr* exhibited an allelopathic but not competitive effect on *CR* when activated carbon was absent. *CR* tuber sprouting was significantly suppressed when sowed in the *BPr* soil. Likewise, *BPr* residue mulch inhibited the *CR* plant density by 87% as compared to natural-occurring *CR* residue mulch in the field. This study revealed that *BPr* might have potential for use as a cover plant and allelopathic mulch to control *CR* in the agroecosystem.

## 1. Introduction

*Cyperus rotundus* L. (*CR*), the most noxious weed on Earth, is a perennial herb in the Cyperaceae family possessing a C4 photosynthetic pathway [1,2]. It was reported that *CR* reproduces rarely by seed but mainly by tubers [3,4]. After sprouting, *CR* can form new tubers within 4–6 weeks and tuber chains in 10 weeks when it grows in a suitable environment [1]. The tuber sprouting within the tuber chain may be inhibited by apical dominance and remains dormant until the rhizomes or chains are cut off [3,4,5]. They then sprout and grow when the aerial parts die due to conventional agricultural practices such as mowing, plowing or herbicide application. Such a superior reproduction ability makes this species invasive and harmful, especially in upland agricultural production systems. Reports have indicated that *CR* can cause a 35–89% reduction in the yields of cabbage, tomato, cucumber, green bean, carrot, okra, bell pepper, onion, garlic and many other crops [6,7,8,9]. According to a study by Bendixen and Nandihalli [2], *CR* is disseminated in over 92 tropical and subtropical countries around the world and causes harvest losses in more than 50 crops.

Plant allelopathy is defined as the direct or indirect effects (positive and/or negative) on plants through the production of allelochemicals. However, the definition of the negative effect is more commonly used in allelopathy research [10,11]. Plant allelopathy has been considered as a herbicide reduction and a labor-saving weed management strategy (compared to hand weeding) in agricultural production systems [12,13]. Viji and Chinnamuthu [14,15] pointed out that the *CR* tuber dormancy could be induced by phenolics such as vanillic acid. Babu and Kandasamy [16] found that the tuber sprouting percentage of *CR* could be inhibited significantly by a water extract of fresh leaves of *Eucalyptus globulus* Labill. Cheema et al. [17] reported that both sorgaab (a sorghum water extract) and sorghum residue mulch could be applied to reducing plant density and shoot dry weight of *CR* when growing maize. Intercropping of sorghum, soybean and sesame for 2 years reduced the *CR* density (70–96%) and dry matter production (71–97%) [18]. Although studies on the application of plant allelopathy to control *CR* are rarely found, the aforementioned results show the potential of using such allelopathy to control the troublesome *CR* weed.

*Bidens pilosa* L. var. *radiata* Sch. Bip. (*BPr*) was studied for its significant allelopathic inhibition effects on many plant species [19,20,21,22]. Xuan et al. [23] indicated that 301 compounds (including polyacetylenes, flavonoids, phenolic acids, terpenes, fatty acids, etc.) have been identified from different parts of *B. pilosa* (*BP*). Among these compounds, the allelochemicals responsible for the phytotoxicity were mainly polyacetylenes and phenolics [19,20]. Campbell et al. [19] found that the phenylheptatriyne (PHT) extracted from the leaves of *BP* significantly inhibited the seedling growth of *Asclepias syriaca* L., *Chenopodium album* L., *Phleum pratense* L., and *Trifolium pratense* L. Deba et al. [20] revealed that phenolics (e.g., pyrocatechin, salicylic acid, *p*-vinylguaiacol, dimethoxyphenol, eugenol, 4-ethyl-1,2-benzenediol, iso-vanillin, 2-hydroxy-6-methylbenzaldehyde, vanillin, vanillic acid, *p*-hydroxybenzoic acid, protocatechuic acid, *p*-coumaric acid, ferulic acid, and caffeic acid) in *BPr* might play important roles in suppressing the germination and seedling growth of *Echinochloa crus-galli* (L.) P. Beauv. and *Raphanus sativus* L. The results of paddy field experiments also demonstrated that phenolic compounds released from the *BPr* residues might cause the death of weeds [24]. On the other hand, it has been reported that *BPr* was capable of exhibiting competitiveness and reducing the crop yields of soybean and corn in both additive and substitutive competition experiments [25]. Wang et al. [26] also indicated that *BPr* significantly suppressed the growth of *Medicago sativa* L. and *Trifolium repens* L. in substitutive competition experiments. It was demonstrated that *BPr* may possess inhibition on the target plants through allelopathy, competition or both. According to our previous experiences, *BPr* was observed being capable of competing with *CR* in areas invaded significantly by *CR* and became dominant afterward. This phenomenon suggested the potential of using *BPr* to control *CR* in an agricultural system. However, to the best of our knowledge, the interaction behind the interspecies interference between *BPr* and *CR* has not yet been clarified. Studies using *BPr* as a cover plant and allelopathic mulch to control *CR* in the agroecosystem are also very scarce. In the present study, it was hypothesized that both allelopathy and competition of *BPr* might be involved in limiting the growth and tuber sprouting of *CR*.

The relationship between plant allelopathy and competition is difficult to separate and/or isolate due to the complexity of the natural environment [27]. Inderjit and del Moral [10] indicated that co-linearities among environmental factors (for example, allelochemical concentration, soil pH, soil moisture, soil organic matter, soil nutrients, and so on) make it difficult to separate the two mechanisms from each other. Moreover, the effort to separate the two mechanisms might induce conditions that would never occur in a natural environment. Therefore, Weidenhamer [27] suggested that to distinguish the two mechanisms is more important and realistic than to separate them. Weidenhamer et al. [28] indicated that resource availability did not alter the predicted slope of the log weight-log density line due to the universal of -1 law in plant ecology, but the phytotoxin such as herbicides and allelochemicals did. Therefore, the density-dependent experiment could be used to distinguish the effect of allelochemicals from the intraspecies competition of the target plant. Besides, due to the strong adsorption capacity for organic chemicals (e.g., phenolic compounds), activated carbon addition was also considered as a feasible approach to distinguish plant allelopathy from the competition without influencing the plant growth [29,30,31].

Accordingly, to test our hypothesis, two pot experiments of density-dependent phytotoxicity and activated carbon addition were conducted to distinguish the allelopathy from the competition between these two investigated species in the semi-natural conditions. Additionally, to assess the potential of using *BPr* as a cover plant and allelopathic mulch to control *CR* in the field, two experiments of the influence of the *BPr* soil and its residues on the tuber sprouting potential and tillers reproduction of *CR* were also conducted in the field.

## 2. Results

### 2.1. Experiment 1: Density-Dependent Phytotoxicity

The inhibition of *CR* seedling growth was observed at a *BPr* residue application rate of 0.1 kg m^−2^ for the treatment of 3 plants pot^−1^, and the shoot, root and total dry weights of *CR* were 72, 51 and 61% of the control (0 kg m^−2^ treatment), respectively (please refer to Table 1 for details). Meanwhile, the inhibition increased with an increasing residue application rate in the 3 plants pot^−1^ treatment. For the treatment of 6 plants pot^−1^, the seedling growth was only inhibited significantly at the application rate of 0.3 kg m^−2^ and the shoot, root and total dry weights of *CR* were 76, 66 and 70% of the control, respectively. There was no significant weight difference among the residue application rates for the 9 plants pot^−1^ treatment. It was noted that the root dry weight per plant in the treatment of 9 plants pot^−1^ was higher than those in the 3 and 6 plants pot^−1^ treatment, implying that the phytotoxicity of the *BPr* residue was diluted due to the higher plant density. In another word, the average phytotoxin absorbed per plant was less at 9-plant treatment than that at lower density treatments.

For each residue application treatment, the ratios of shoot, root and total dry weight to the control were increased from low density (3 plants pot^−1^) to high density (9 plants pot^−1^), and the growth inhibitions were higher at low than at high plant density treatments. The result also implied the presence of phytotoxin in the residue of *BPr* and showed that the response of *CR* seedling growth to the residue of *BPr* was density-dependent.

The shoot to root ratios of *CR* in the 3 plants pot^−1^ treatment with *BPr* residue were significantly higher compared to the control. The ratios were 1.25, 1.32 and 1.36 of the application rates of 0.1, 0.2 and 0.3 kg m^−2^ treatments, respectively (as shown in Table 2). The ratio greater than 1 indicated that the root growth was more suppressed by the residue of *BPr* than the shoot growth, and the biomass allocation pattern was altered. For the 6 and 9 plants pot^−1^ treatments, the shoot to root ratios of *CR* at the application rates of 0.2 and 0.3 kg m^−2^ were higher than that of the control but these ratios were smaller than 1. The influence of the residue of *BPr* on the shoot to root ratio of *CR* also showed a density-dependent phytotoxic effect. The ratios decreased with an increase in plant density for all the investigated application rates, also showing that the phytotoxin was diluted by the increasing density.

The residue of *BPr* also demonstrated significant inhibition on the *CR* tuber numbers per plant at the low-density treatment (i.e., 3 plants pot^−1^). The tuber numbers per pot were 69, 58 and 71% of the control at the 0.1, 0.2 and 0.3 kg m^−2^ residue application rates, respectively (Table 3). For the higher density treatments (i.e., 6 and 9 plants pot^−1^), the inhibition effect of the residue on the tuber number per plant was reduced. Hence, the inhibition of tuber number by the phytotoxin in the residue was density-dependent. However, the tiller numbers per plant of *CR* were slightly reduced by the increasing residue application rate, indicating that the residue had less inhibition effect on the tuber sprouting than that of the density effect.

**Figure 1:**
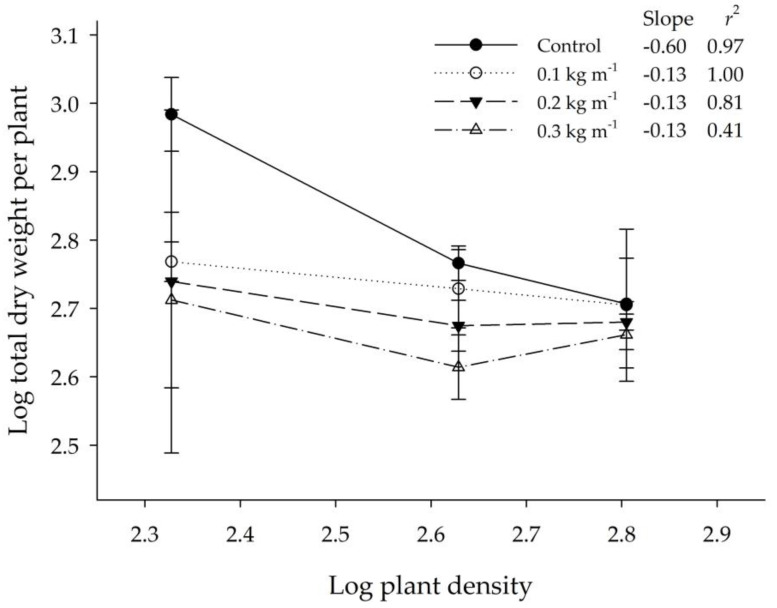
Relationship of log mean total dry weight and log plant density of *C. rotundus* (*CR*) for the residue of *B. pilosa* var. *radiata* (*BPr*) application at four different rates.

The relationship of log total dry weight per plant vs. log plant density showed that the regression slopes of the investigated treatments of *BPr* residues deviated from that of the control and the deviation became more apparent as the residue application rate increased (Figure 1). The different reduction between the low and high density treatments showed again that the *CR* absorbed more phytotoxin per plant at the low density treatment (3 plants pot^−1^) than at the high density treatments (6 and 9 plants pot^−1^). Besides, the results also demonstrated that the plant growth might be stimulated at high density due to hormesis (stimulation at subtoxic concentration) at the 9 plants pot^−1^ treatment [11].

### 2.2. Experiment 2: Interspecies Competition between BPr and CR

**Figure 2:**
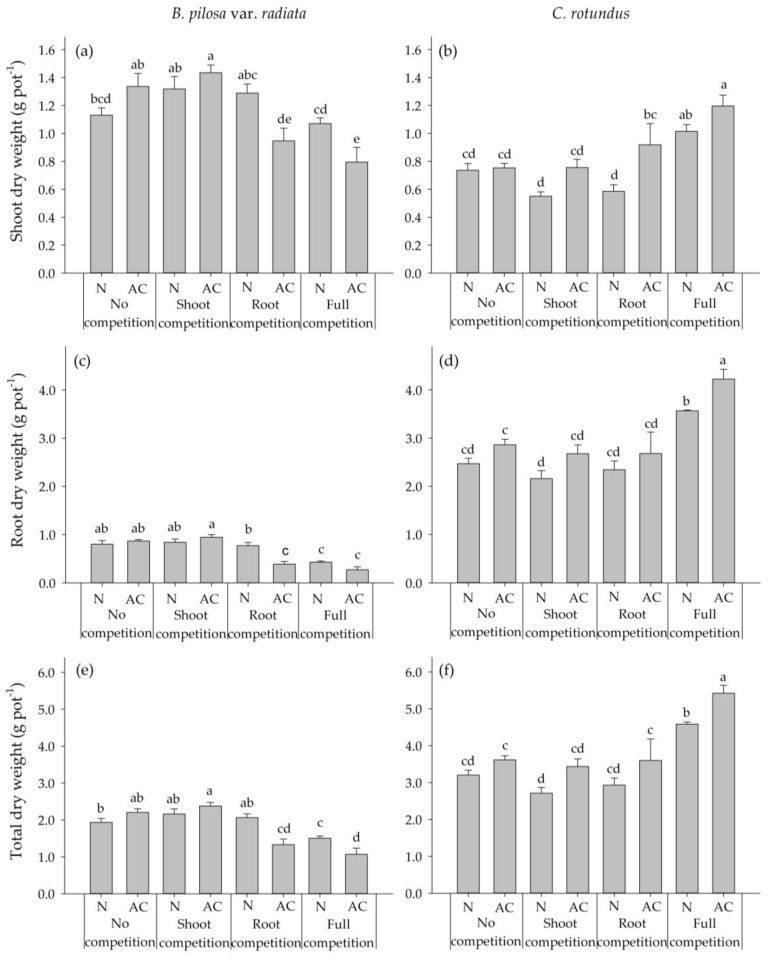
Shoot, root and total dry weight per pot of *BPr* and *CR* when grown with two activated carbon application treatments and subjected to four competition modes. (**a**) Shoot dry weight of *BPr*. (**b**) Shoot dry weight of *CR*. (**c**) Root dry weight of *BPr*. (**d**) Root dry weight of *CR*. (**e**) Total dry weight of *BPr*. (**f**) Total dry weight of *CR*. AC and N denoted pots added with and without activated carbon application, respectively. Error bars are the standard error of mean (*n* = 5). Means with the same letter(s) are not significantly different at 5% level by LSD test.

In experiment 2, through the relative arrangement of aboveground and belowground partition coupled with the disposition of *BPr* and *CR*, four competition modes were obtained: (1) both the shoot and root of the two species were separated (NO competition), (2) only the root was separated (SHOOT competition), (3) only the shoot was separated (ROOT competition) and (4) neither the shoot nor root was separated (FULL competition). By comparison between the results of the NO and SHOOT competitions, it was found that the shoot dry weights of *BPr* and *CR* were higher with the activated carbon treatment (AC treatment) than without activated carbon (N treatment), but only the difference in SHOOT competition of *CR* was significant (Figure 2a,b). For the ROOT competition, the shoot dry weight of *BPr* was reduced by 32% at AC treatment as compared to N treatment. *CR* showed an adverse response to the activated carbon, and the shoot dry weight was enhanced by 44% at AC treatment as compared to N treatment. Compared to the other three competitive experiments, *BPr* exhibited the lowest shoot weight at the N (1.07 g pot^−1^) and AC (0.79 g pot^−1^) treatments in the FULL competition, while *CR* produced the highest shoot weight at the N (1.01 g pot^−1^) and AC (1.2 g pot^−1^) treatments in the FULL competition.

*CR* had a higher root growth than *BPr* for all tested competitions. Similar to the shoot growth, the root growth of *CR* was stimulated by the AC treatment in the NO and SHOOT competitions, and only the difference in SHOOT competition of *CR* was significant (Figure 2c,d). For the ROOT competition, the root dry weight of *BPr* was significantly reduced by 50% in AC treatment compared to N treatment. By contrast, the root growth of *CR* was 25% higher in AC treatment than in N treatment. 

Comparing all the competitions showed that *BPr* had the lowest root dry weight (0.43 g pot^−1^ for N treatment and 2.27g pot^−1^ for AC treatment) in the FULL competition, whereas *CR* had the highest root dry weight for each N (3.57 g pot^−1^) and AC (4.23 g pot^−1^) treatment in the FULL competition.

After four weeks of growth, *CR* accumulated more total biomass than *BP*r in all the treatments Figure 2e,f. However, for all the competitions except for the NO competition, the growth reduction of *CR* at N treatment implied that *BPr* might possess the allelopathic effect through shoot leachate and root exudation. Meanwhile, under the ROOT and FULL competitions, the total growth reduction of *BPr* at AC treatment might be caused by the aggressive resource competition of *CR* root.

**Figure 3:**
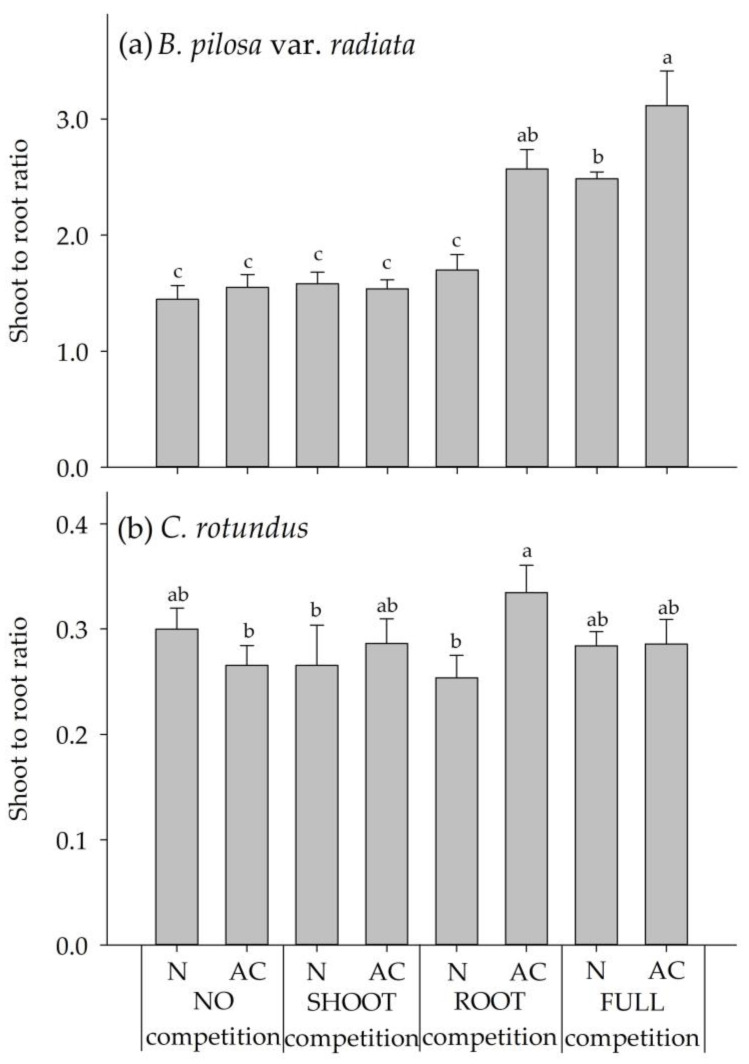
Shoot to root ratio of *BPr* and *CR* when grown with two activated carbon application treatments and subjected to four competition modes. (**a**) Shoot to root ratio of *BPr*. (**b**) Shoot to root ratio of *CR*. AC and N denoted pots added with and without activated carbon application, respectively. Error bars are the standard error of mean (*n* = 5). Means with the same letter(s) are not significantly different at 5% level by LSD test. Data were log-transformed prior to analysis.

Although both plants are perennial herbs, they had opposite biomass allocation patterns. The biomass distribution was facilitated firstly in shoot rather than in root for *BPr,* but such distribution occurred in root rather than in shoot for *CR* at the growing stage. With the NO and SHOOT competitions, neither N nor AC treatment had an effect on the shoot to root ratios of *BPr* and *CR*. The average shoot to root ratios of *BPr* and *CR* in these competitions were 1.5 and 0.28, respectively (Figure 3). 

**Figure 4:**
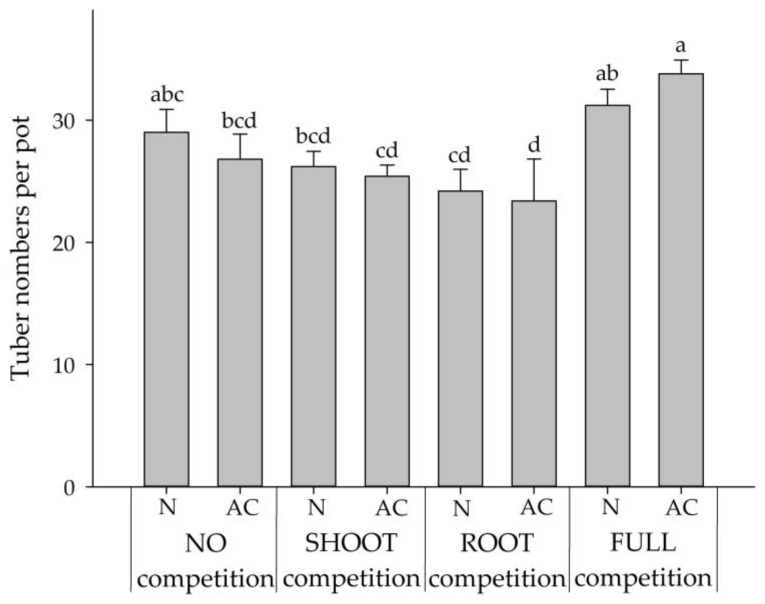
Tuber numbers per pot of *CR* when grown with two activated carbon application treatments and subjected to four competition modes with *BPr*. AC and N denoted pots with and without activated carbon application, respectively. Error bars are the standard error of mean (*n* = 5). Means with the same letter(s) are not significantly different at 5% level by LSD test. Data were square-root transformed prior to analysis.

For the ROOT competition, the shoot to root ratios were significantly increased at AC treatment for both *BPr* and *CR*. The increased ratio of *BPr* was caused by the strong reduction of the root growth (Figure 2c and Figure 3), while the ratio of *CR* increased due to the thriving growth of the shoot (Figure 2b and Figure 3). Under the FULL competition, the shoot to root ratios of *BPr* were significantly higher at both activated carbon treatments than that in the control (NO competition), whereas the ratios of *CR* were shown no difference from that in the control. Similar to the ROOT competition, the reduction of *BPr* root growth under the FULL competition resulted in the increased ratios. However, the unchanged ratios of *CR* under the FULL competition might result from the thriving growth of both the shoot and root (Figure 2b,d and Figure 3). The *CR* tuber proliferated greatly in all treatments after ten weeks of growth (Figure 4). Different from the responses of *CR* seedling growth to the *BPr* allelopathy, the N treatment showed no inhibition on the tuber proliferation in all the competitions when compared with the AC treatment.

### 2.3. Experiment 3: The Tuber Sprouting of CR in the Field of Mature BPr Vegetation

The response of *CR* tuber sprouting to the presence of *BPr* showed that the highest tuber sprouting percentage, mean sprouts per quadrat and dry weight per sprout, occurred in the treatment mulched by the opaque plastic sheet (OP treatment), followed by sowing tubers in the *BPr* vegetation without (VN treatment) and with removing the shoots and litter (VS treatment) (Table 4). The OP treatment here was considered as a control measure because it was a common weed controlling manner in agricultural practice. It was observed that it had a high sprouting percentage of 81% with 30.75 sprouts per quadrat and a mean dry weight per sprout of 9.98 mg. The tuber sprouting percentage (52%), mean sprouts per quadrat (18 sprouts per quadrat) and dry weight per sprout (5.26 mg) were decreased when the tubers of *CR* were sowed in the *BPr* soil with removing shoot and litter (VN treatment). The results of VN treatment indicated that although the shoots and litter of *BPr* were removed, the allelochemicals remained in the soil and continued to inhibit the *CR* tuber sprouting. On the other hand, only one tuber with one sprout in total was found in VS treatment, demonstrating that the presence of the living *BPr* might continue to release allelochemicals to inhibit the tuber sprouting of *C. rotundus*.

### 2.4. Experiment 4: The Effects of Vegetation and Residue Mulch of BPr on the Reproduction of CR

At the beginning of the experiment (0 days after sowing, 0 DAS), the tuber densities of the plots covered with *BPr* and *CR* were 55.51 and 60.44 tubers dm^−2^, respectively, and the dry weights per tuber were 98.41 and 101.12 mg tuber^−1^, respectively (Table 5). No significant difference was found in the tuber density and the dry weight per tuber between the two tested cover plants before starting the experiments by sowing with the seeds of *BPr*. After the seeds were sowed, it was observed that although most seeds germinated in the first week, *CR* reproduced and grew more rapidly than *BPr* seedlings at the early stage. The seedlings of *CR* continued growing even the canopy of *BPr* was closed. Therefore, the tuber density increased approximately two folds for both the cover plant treatments with *BPr* (92.5 tubers dm^−2^) and *CR* (104.91 tubers dm^−2^) on the 69 DAS and no significant difference was found between them. However, the dry weight per tuber at the cover plant of *BPr* (49.51 mg tuber^−1^) was significantly lower than that of *CR* (63.31 mg tuber^−1^), indicating that the shadow and/or allelopathy of *BPr* might reduce the tuber biomass accumulation.

**Figure 5:**
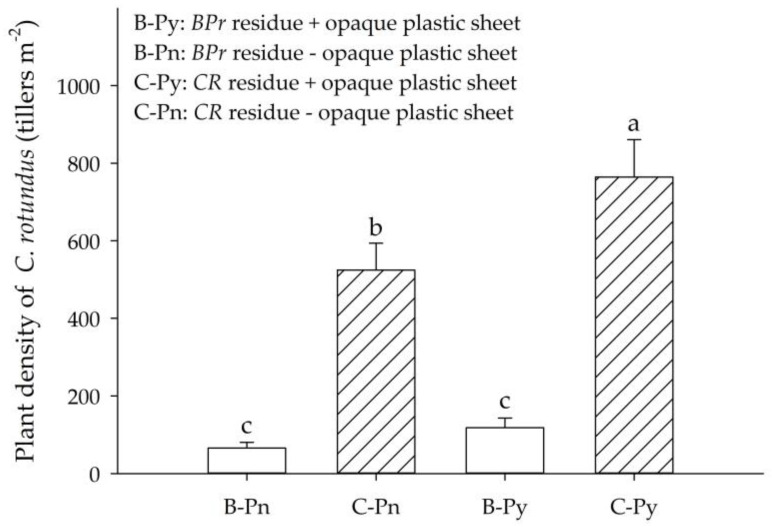
The influence of *BPr* as a cover plant on the regeneration of *CR*. Plant density of *CR* was investigated two weeks after mowing (69 DAS). Error bars are the standard error of mean (*n* = 6). Means with the same letter(s) are not significantly different at 5% level by LSD test.

**Figure 6:**
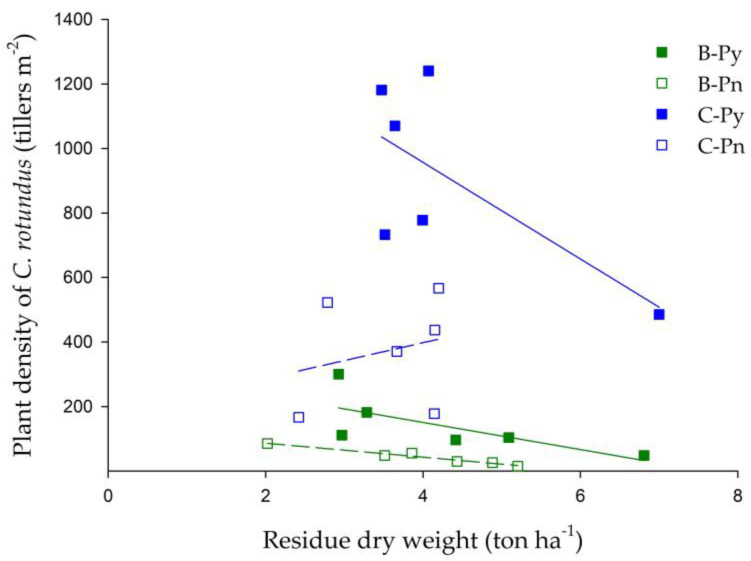
Relationship of plant density of *CR* (tillers m^−2^) and residue dry weight (ton ha^−1^). B-Py is *BPr* residue covered with the opaque plastic sheet, B-Pn is *BPr* residue covered without the opaque plastic sheet, C-Py is *CR* residue covered with the opaque plastic sheet and C-Pn is *CR* residue covered without the opaque plastic sheet.

Both of the cover plant treatments were subject to mowing at 69 DAS and all the plant residues were left on their plots. Afterward, for the cover plant of *BPr*, half of the plots were mulched with the opaque plastic sheet (B-Py treatment) and another half were not (B-Pn). The same handling was conducted for *CR*. C-Py and CPn treatments denoted the plots mulched with and without the opaque plastic sheet, respectively. Two weeks afterward (i.e., 83 DAS), the investigation of the *CR* plant density showed that the highest *CR* density was found at C-Py treatment (764 plants m^−2^), followed by C-Pn (524 plants m^−2^), B-Py (118 plants m^−2^) and B-Pn (65 plants m^−2^) treatments (Figure 5). According to the results, the treatments grew with *BPr* and mulched with its residues (B-Py and B-Pn treatments) significantly inhibited the reproduction of *CR* as compared to that grew without *BPr* and mulched with the residues of *CR* (C-Py and C-Pn treatments). The results also further indicated that the reproduction of *CR* was suppressed by the allelopathy of *BPr* rather than the opaque plastic sheet. In short, the relationship between the residue dry weight and *CR* plant density illustrated that the *BPr* residues could provide suppression on the density of *CR* even at a lower quantity of 2 ton ha^−1^ (Figure 6); meanwhile, the *CR* density was suppressed by 87% on average at the mean application rate of 4 ton ha^−1^ (Figure 5 and Figure 6).

## 3. Discussion

### 3.1. Distinguishing Allelopathy from Competition

Studies indicated that the leachate or residues of allelopathic plants might inhibit the growth of *CR*. For example, the leachate of *E. globulus*. fresh leaves significantly reduced both the shoot and root dry weight of *CR* [16]. Residues of *Helianthus annuus* L., *Sorghum bicolor* (L.) Moench and *Brassica campestris* L. reduced the plant density and dry weight of *CR* [32,33]. However, due to the complexity of the ecological environment, it is hard to distinguish the allelopathy of the leachate or residues from the competition (including intraspecies or plant-microbial interactions) [27,34].

Weidenhammer et al. [28] firstly provided experimental evidence that density-dependent phytotoxicity of allelochemicals could be used to distinguish allelopathy from the intraspecies competition (or other microbial activities). Allelochemicals could cause a greater growth reduction on the target plant at low density than that at high density due to the dilution of phytotoxicity of allelochemicals. Besides, Weidenhammer et al. [27,28] also pointed out that the slope of log mean plant weight versus log mean density would be altered due to interactions between density and allelopathy, thus deviated from the expected log yield-density relationships of the -1 law of plant ecology [27]. In the present study, the results of experiment 1 demonstrated that the phytotoxicity of *BPr* residues to the shoot and root (including tuber numbers) growth of *CR* seedlings was density-dependent (Table 1 and Table 3). Residues exhibited the greatest phytotoxicity to the seedlings at the low density (3 plants pot^−1^) and the reduction of growth decreased as the density increased. Simultaneously, the slopes of the log mean plant weight versus log mean plant density curves at the tested residue application rates obviously deviated from that of the control (Figure 1). This also provided evidence for the existence of density-dependent phytotoxicity in the residue of *BPr*.

Scavo et al. [13] indicated that a two-way relationship might exist between soil characteristics and allelochemicals that affects the retention, transport and transformation processes of allelochemicals in soil. In a short-term pot experiment, although the soil organic matter content, available nitrogen, pH and EC were altered by adding allelochemical residues in the soil, the phytotoxicity of the *Chenopodium murale* L. residues possessed the main negative effects on the chickpea and pea [35]. Based on the findings of these two studies, the soil organic matter content, available nitrogen and EC in our study were supposed to be raised with the increase in *BPr* residue application rate and stimulated the growth of *CR.* However, the negative growth response of *CR* demonstrated that the allelopathy of *BPr* residues might be the main cause of the influence of this experiment.

It has been reported that intercropping of allelopathic plants in cotton improved the cotton yield and reduced the *CR* population [18]. However, little has been investigated regarding the effect of interspecies competition and allelopathy at the same time due to the difficulties of distinguishing them from each other under natural or semi-natural conditions. In experiment 2, activated carbon was added to four different competition modes to absorb the allelochemicals in the pots. The findings showed that under SHOOT competition, neither the growth of *BPr* nor *CR* was suppressed from each other with the addition of activated carbon (AC treatment) as compared to the AC treatment under NO competition. However, the shoot and root growth of *CR* was reduced at N treatment (Figure 2). It was presumed that the growth reduction of *CR* at N treatment under SHOOT competition might result from the phytotoxicity in the shoot leachate of *BPr*. Besides, notwithstanding that light is a considerable factor for shoot competition, no evidence supported that either *BPr* or *CR* suffered shading from the other species. Previous literature also indicated that light is not an important factor for shoot competition before plant canopies are capable of shading on another species [36].

Under ROOT and FULL competitions, the growth of *CR* was enhanced while *BPr* was decreased at AC treatment as compared with N treatment. Researches indicated that *CR* often demonstrated a predominant root competition with coexisting plants. Tuor and Froud-Williams [37] showed that *CR* could significantly decrease the shoot dry weight and height of both maize and soybean under root and full competitions as compared to zero competition (the same as NO competition in this study). Horowitz [38] indicated that the growth of citrus seedling was significantly inhibited by *CR* despite of fertilizing with nitrogen or not. He further supposed that the phytotoxic substances produced by *CR* might partly contribute to the competition with citrus. Although the previous studies demonstrated that *CR* could compete with coexisting plants through allelopathy, the allelopathic effects of *CR* on *BPr* were not observed in experiment 2 since the *CR* growth was suppressed by *BPr* when no activated carbon was applied.

The most conflicting results from experiments 1 and 2 were the comparison of the shoot to root ratios. The result of experiment 1 indicated that both the plant density and residue application rate of *BPr* altered the ratios but in opposite directions (Table 2). The ratios decreased as the plant density increased for a given residue application rate but increased with the increase of application rate. According to the prediction of optimal partitioning theory (OPT), plants tend to partition more biomass in root than in shoot when major nutrients are deficient and thus result in a lower shoot to root ratio [39]. A similar tendency reported by Williams et al. [40] showed that *CR* allocated more biomass to root rather than shoot when growing at high density. The effect of plant density on the ratios appeared that *CR* in the high density subjected to more resource competition and the nutrient supply for each plant was reduced. However, the response of shoot to root ratio was inverse in trend by the application of *BPr* residues in each given density treatment. It was presumed that the root nutrient absorption ability of *CR* was impaired due to the deleterious effects of *BPr* allelochemicals such as the phenolic acids thus resulted in increasing the shoot to root ratio [41,42].

In experiment 2, under the competition including root interference (i.e., ROOT and FULL competitions), both *BPr* and *CR* had statistically-significant lower shoot to root ratios in the presence of allelopathy (N treatment) than that in the absence (AC treatment) except for the *CR* under FULL competition. The responses of shoot to root ratio of *CR* to the allelopathic effects of *BPr* roots did not increase as that influenced by the residues of *BPr* in experiment 1. Two possibilities were supposed for the difference between experiments 1 and 2. First, the allelochemicals in the residues and the root exudation might be different. Deba et al. [20] found that the main phenolic compounds in the leaves, stems and roots of *BPr* were similar in composition but different in content. Second, the allelopathic effects coupled with root competition caused a different inhibition mechanism in experiment 2. A similar result from Nilsson [30], who reported that Scots pine seedlings had a lower shoot to root ratios when in the presence of both allelopathy and interspecies root competition. Schenk [42] pointed out that plant roots exhibited different responses when overlapping with ‘self’ and ‘non-self’ roots, and the responses might further be altered by allelochemicals that inhibited root growth.

El-Rokiek [43] pointed out that the phenolics (ferulic acid, caffeic acid for example) in the mango leaves might inhibit the seedling growth and tuber sprouting of *CR*. Fifteen phenolic compounds isolated from *BPr* were also reported to possess the allelochemicals of phenolics (e.g., caffeic, ferulic, *p*-coumaric, *p*-hydroxybenzoic, salicylic acid and so on) [20,23]. Such phenolics were reported to cause deleterious damage to plant roots. For example, Einhellig [44] indicated that the salicylic acid appeared to cause damage to the membrane structure and permeability of the root cell while the caffeic acid decreased the nitrogen, phosphorus, potassium, iron and molybdenum in cowpea. Bergmark et al. [41] showed that ferulic acid inhibited the nitrogen uptake in the roots of maize seedling. Abenavoli et al. [45] also demonstrated that the trans-cinnamic, ferulic and *p*-coumaric acid reduced the net nitrogen uptake and plasma membrane H^+^-ATPase activity. PHT, a putative allelochemical of polyacetylene, was presumed to release phytotoxic radicals and this inhibitory mechanism could be enhanced by illuminating with sunlight or near-UV light. PHT was found to exist in the leaves of *B. pilosa* and was reported to suppress the seedling growth of *A. syriaca*, *C. album*, *P. pratense* and *T. pratense* with LC_50_ of 0.66, 0.83, 2.88 and 1.43 ppm, respectively [19]. In experiments 1 and 2, *BPr* residues, shoot leachates and root exudates were supposed to release phenolics and PHT to the soil and interfered with the growth of *CR*. Besides, it was also reported that allelopathic plants exhibited higher inhibitory effects on the neighboring plants when growing under nutrient-deficient conditions [13]. Therefore, since no extra fertilizer was added in experiment 2 during the ten-week growing period, the nutrient-deficient conditions might also contribute to stimulating the allelochemical exudation of *BPr* when competing for nutrients with *CR* (under ROOT and FULL competitions).

### 3.2. The Influence of B. pilosa var. Radiata on the Reproduction of CR in the Field

The *BPr* soil exhibited a strong phytotoxicity effect on the tuber reproduction of *CR*. The tuber sprouting percentage, mean sprouts per quadrat and dry weight per sprout were lower in both the VN (with removing the shoots and litter) and VS treatments (without removing the shoots and litter) than those in the OP treatment. Moreover, the VS treatment possessed higher suppression than VN treatments (Table 4). In a natural environment, the concentration, movement and persistence of allelochemicals determine the phytotoxic level of donor plants on the target plants [13]. The water-soluble allelochemicals such as the phenolics were considered to have a short residence time in the soil due to the rapid leaching and degradation [46,47]. In the present study, the results of experiments 1 and 2 illustrated that *BPr* might release allelochemicals through its residues, shoot leachates and root exudes. The removal of aboveground *BPr* and litter in the VN treatment interrupted the input of allelochemicals (e.g., phenolics and PHT) into the soil. The remaining phytotoxicity of *BPr* soil was expected to degrade soon when no allelochemical was continuously released from the aboveground plant and litter to the quadrat. Meanwhile, the replenishment of allelochemicals from *BPr* plants outside the quadrat was also limited due to the slow rates of chemical diffusion in soil, sorption of the soil particles and organic matter, and microbial degradation [48].

In the field severely invaded by *CR*, the tubers proliferated obviously in both cover plant treatments during the experimental period (Table 5). It was supposed that the apical dominance in tubers was broken off when the tuber chains were cut off due to the plowing before the experiment [4,5], and a large number of dormant tubers in chains started sprouting. Likewise, with the characteristics of the C4 photosynthetic pathway [1], the sprouts of *CR* grew faster than the seedlings of *BPr*. Hence, the tuber numbers increased in all treatments before mowing.

The inhibition effects of the allelopathic residues on the reproduction of *CR* depended on plant species and approaches used. Mahmood and Cheema [49] reported that soil-incorporated sorghum stalks (15 ton ha^−1^) had less inhibitory effect on the density of *CR* than the surface-applied treatment (15 ton ha^−1^). Khaliq et al. [32] further indicated that the residue combination of sorghum, sunflower and brassica (each at 7.5 ton ha^−1^) reduced the *CR* plant density by 87% as compared to the control. On the contrary, the soil-incorporated allelopathic straws of wheat and rye could efficiently decrease the weed densities of *Portulaca oleracea* L., *Amaranthus retroflexus* L. and *Echinochloa colonum* L. but failed to inhibit the emergence of *CR* [50]. In the present study, despite the fact that the tubers proliferated before the cover plants were mowed, the plant density of *CR* was significantly reduced in the presence of *BPr* residue mulch and decreased with the increasing residue dry weight. In addition, it was also found that the *CR* plant density was higher in both cover plant species in the presence of opaque plastic sheet treatment as compared to that in the absence of the opaque plastic sheet. Although soils covered with the plastic sheet was observed to elevate the temperature in the air space (between the sheet and soil surface) and the upper soil [51], it was found that no negative effect on the tuber reproduction unless the upper soil temperature was elevated up to 45°C and persisted for more than 7 h per day [52]. Moreover, studies indicated that phenolic compounds released from apple residues decreased with the elevating soil temperature [53]. Meanwhile, due to the characteristics of photosensitization and high activity, the phytotoxicity of polyacetylenes such as PHT might be reduced in the darkness and the temperature higher than 30 °C [19,54]. Therefore, in this experiment, the allelochemicals of *BPr* might degrade more rapidly in the B-Py and C-Py treatments than in the B-Pn and C-Pn treatments due to the elevating temperature under the sheet.

Plant species that possess great competitiveness and invasive capacity with crops or native species usually have a strong allelopathic capacity [55]. *BP*, especially its variety *BPr*, was found to be highly invasive in subtropical and tropical regions [23]. Field competition studies conducted by Ng et al. [56] showed that *BPr* was dominant in groups consisting of Poaceae and C3 plants in competition with monoculture and polyculture groups of sixteen species. Likewise, *BP* and *BPr* have been found to possess phytotoxic effects on its sympatric plant species, such as *Bidens bipinnata* L. and *Pteris multifida* Poir., in the ecosystems [21,22]. In southeast Asia, *BP* and *BPr* have been investigated for their capability in paddy weed management. For example, Hong et al. [57] evaluated ten allelopathic species for paddy weed control in Vietnam and found that *BP* was an effective allelopathic species to eradicate 80% more of weeds and increase rice yield more than 20%. Krumsri et al. [58] examined the phytotoxic effects of *BP* residue on *E. crus-galli* under various conditions, and found that fresh *BP* residues exerted more phytotoxicity than the dried residues. Meanwhile, both soil mulching and incorporating with *BP* residues significantly decreased the density of *E. crus-galli* when using the *BP* plants harvested at a 60-day growth stage. In Thailand, Poonpaiboonpipat and Poolkum [24] indicated that the most effective application rate of *BPr* residue in the paddy field was 4 ton ha^−1^ which inhibited the weed growth by 86.73% and increased the rice yield by 81.03%. These findings support the fact that the residues of *BP* and *BPr* have been successfully used as natural herbicides in the weed management of paddy fields and provided additional benefits to rice yields. In this study, the allelopathic effects of *BPr* in the upland agricultural system were studied in the open fields. Given the results of field experiments, with the contribution of phytotoxicity of allelochemicals, *BPr* exerted great competitiveness on the noxious weed of *CR* in the field. B-Pn and B-Py treatments attained 87% inhibition of *CR* plant density as compared to the results of C-Pn and C-Py treatments.

Additionally, some research further showed the benefits of preventive weed control measures by introducing allelopathic species into the crop rotation. Scavo et al. [59] indicated that the field rotated with an allelopathic crop of *Cynara cardunculus* L. for three years showed a significant decrease (34–50%) in the amount of soil weed seeds when compared to the traditional wheat/faba bean rotation. In the present study, the results showed that soils in a three-year-old *BPr* vegetation exerted strong allelopathic effects on *CR* tuber sprouting, especially in VS treatment.

In addition to an invasive weed, *BP* is also an edible and medicinal herb in many countries and has been extensively investigated for the potential of pharmacological use [23]. The good agricultural practice of *BP* was established because of its frequent pharmacology application in Taiwan [60]. Hence, this species should not only be regarded as an invasive weed but also a medicinal crop. However, for further introducing *BPr* into the crop rotation systems or using its residue as allelopathic mulch in weed managements, its allelopathic effects on the subsequent or the standing crops require additional consideration. Ng et al. [56] reported that some legume species such as *Phaseolus radiatus* L. *Mimosa pudica* L. and *Sesbania cannabina* (Retz.) Poir. were observed to be more resistant to *BPr* invasion than *Ipomoea aquatica* Forssk. and *Zea mays* L. Wang et al. [26] indicated that the forage legume, *Vicia villosa* Roth, exhibited a greater competitive ability against *BP* than *M. sativa* and *T. repens* in the competition experiment. Although Tembo et al. [61] indicated that *BP* extracts exerted little benefit for pest control in three legume crops, no growth or yield inhibition was observed. These findings indicated that the inhibitory effects of *BPr* should be species-dependent, and some legumes might be the proper candidate species in the crop rotation with *BP*. Our results of experiment 3 also indicated that the phytotoxicity decreased obviously after the aboveground plant materials were removed for two weeks. Therefore, it was confirmed (1) the field allelopathic potential of *BPr* in monoculture for medicinal herb cultivation, regardless of its growth in a field severely invaded by *CR* and (2) the possibility of introducing *BPr* within an upland crop rotation for the weed control and reduction in herbicide utilization. However, the applications of *BPr* as a rotation crop and/or allelopathic mulch in the agricultural practices deserve another future study.

## 4. Materials and Methods

### 4.1. Plant Material Preparation

Seeds and plant residues (including leaves, stem, flower and seeds) of *BPr* were collected from the plants grown in the experimental field of Taitung District of Agriculture Research and Extension Station (Taitung DARES, located at 22°44′52″ N, 121°8′59″ E). Seeds were stored in a paper bag at room temperature before use. The plant residues of the bloom stage used were harvested and dried at ambient temperature for 15 days. The dried residues were crushed into small pieces (<2 cm) with an electric cutter, and stored at −20 °C before use. Tubers of *CR* were collected from the experimental field of Taitung DARES. Tubers with a diameter of 0.5–1.0 cm were chosen and washed for use on the same day when the experiment started.

### 4.2. General Experimental Design

The present study consisted of four experiments. Experiment 1 aimed to distinguish the *BPr* residue allelopathy from the intraspecies competition of *CR* by evaluating the density-dependent phytotoxicity. Experiment 2 was designed to explore the presence of allelopathy by adding activated carbon and the competitivity of the two investigated species. In experiment 3, the *CR* tubers were sowed in a three-year-old *BPr* field with or without removing the aboveground plant materials. The results of experiment 3 helped to recognize if the phytotoxicity of *BPr* changed after mowing. Experiment 4 assessed the effects of using *BPr* as a cover plant and mulch on the reproduction of *CR*.

The first two experiments were performed in semi-natural conditions to provide evidence of the presence of allelopathy of *BPr*. The last two experiments conducted in the field helped to assess the potential of using *BPr* to control *CR* in the field. The results from the four experiments were compared to show the difference in observation between greenhouse and field experiments.

### 4.3. Experiment 1: Density-Dependent Phytotoxicity

Soil collected from the vegetable farmland invaded severely by *CR* in Taitung DARES was air-dried, sieved through 2-mm mesh to remove large plant debris. Soils of 1.2 kg mixed thoroughly with four application rates of *BPr* residues (0, 1.4, 2.8 and 4.2 g pot^−1^) were placed in a 13.5 cm diameter plastic pot. The levels of 0, 1.4, 2.8 and 4.2 g pot^−1^ were equivalent to 0, 0.1, 0.2 and 0.3 kg m^−2^, respectively. Tubers of *CR* were pre-sprouted before the experiments. The densities of 3, 6 and 9 sprouted tubers were planted for low-, medium- and high-density treatments, respectively. Experiment 1 was carried out in a greenhouse of Taitung DARES with two factors complete random design (CRD). Each combination (residue application rate × plant density) was repeated four times. All pots were watered as needed but no fertilizer was added during the experiment. After four weeks, plants were harvested, divided into shoot and root, and dried for 48 h at 80 °C. The dry weight of shoot and root, as well as the numbers of tuber and tiller were determined.

### 4.4. Experiment 2: Interspecies Competition between B. pilosa var. radiata and C. rotundus

**Figure 7:**
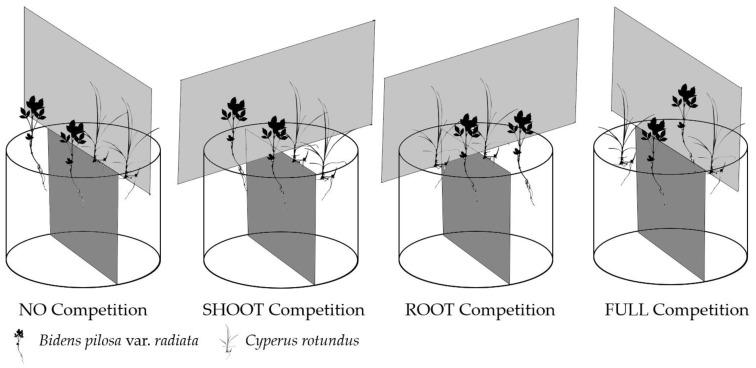
The design of the interspecies competition experiment. The relative arrangement of aboveground and belowground partition and the disposition of two plants were designed to give four modes of competition (i.e., NO, SHOOT, ROOT and FULL competitions).

The method used in the interspecies competition was modified from the experimental protocols proposed by Snaydon [62]. A plastic pot (13.5 cm in diameter and 13 cm in depth) was equally subdivided by a plastic plate to serve as the belowground partition. The plastic plate was sealed with neutral silicone gel to the sides and base of the pot. Another plastic plate of 30 × 30 cm fixed vertically on the upper edge of the pot to serve as the aboveground partition. The relative arrangement of aboveground and belowground partition coupled with the disposition of *BPr* and *CR* were designed to compare various competitions, i.e., NO, SHOOT, ROOT and FULL competitions (Figure 7). 

For each competition, a 1.2 kg soil mixed thoroughly with (AC treatment) or without (N treatment) fine powdered activated carbon (pure grade) at the ratio of 50 to 1 was placed evenly in the two subdivisions of each pot. All pots were sprayed with R.O. water (50 mL per pot) per day and watered as needed but no fertilizer was added during the experiment. Experiment 2 was also conducted in the greenhouse of Taitung DARES with two factors complete random design (CRD). Each combination (competition × activated carbon addition) was repeated five times.

### 4.5. Experiment 3: The Tuber Sprouting of CR in the Field of Mature BPr Vegetation

To prevent the interference of the invaded *CR*, the field tuber sprouting experiment was carried out in a strip of three-year-old vegetation of *BPr* (24 m in length and 1.5 m in width) and its adjacent fallow field in Taitung DARES in January of 2020. The strip of *BPr* had been inspected before the experiment to assure no invasion of *CR*. Eight 30 cm × 30 cm quadrats were set randomly in the *BPr* strip. Half of the quadrats was selected to remove the aboveground plant shoots and litter to make the surface bare (VN treatment). Another half were left for shade (VS treatment). Four 1 m × 1 m plots were randomly selected in the adjacent bare ground (approximately 3 m far from the *BPr* strip). Weeds included tubers of *CR* in the plots were carefully removed by hand weeding. For each plot, one 30 cm × 30 cm quadrat was set in the center and mulched with an opaque plastic sheet (OP treatment). As a reference treatment with common weed management practice, the OP treatment was conducted to compare the tuber sprouting with VN and VS treatments. Twenty-five tubers (0.5–1.0 cm in diameter) of *CR* were sowed in each quadrat. Sprouted tubers, sprouts per quadrat and dry weight per sprout were explored two weeks after sowing.

### 4.6. Experiment 4: The Effects of Vegetation and Residue Mulch of BPr on the Reproduction of CR

In the winter of 2019, a field seriously invaded by *CR* was chosen for the present study and divided into 24 plots (3 m in length and 1 m in width). Each plot was separated by a 0.8 m wide ditch. On 17 October 2019, three soil cores (2 inches in diameter and 15 cm in depth) per plot were sampled to investigate the numbers and dry weight (dried for 48 hr at 80 °C) of *CR* tubers in the upper 15 cm of soil. All the plots were mowed and the seeds of *BPr* (8 g polt^−1^) were sowed randomly in half of the plots on the following day (18 October 2019). *CR* was allowed to grow in another twelve plots without sowing *BPr*. On 26 December 2019 (69 DAS), all the aboveground plant parts were mowed, the plant residues were weighted and left on the plot. After mowing, three soil cores per plot were sampled again for investigating the numbers and dry weight of *CR* tubers in the upper 15 cm of soil. Half plots of *BPr* (B-Py) and *CR* (C-Py) were mulched with an opaque plastic sheet while another half plots of *BPr* (B-Pn) and *CR* (C-Pn) were not mulched. The tiller numbers of *CR* of the aforementioned four treatments (B-Py, C-Py, B-Pn and C-Pn) were counted two weeks after mowing. Experiment 4 was conducted in two factors complete random design (CRD) with 6 replications for each combination (plant species × opaque plastic sheet mulch).

### 4.7. Statistical Analysis

Levene test was used to test for homogeneity of variance. The experimental data were subjected to analyses of variances (ANOVA) and Fisher’s LSD post-hoc test by the SAS software (SAS Enterprise Guide 7.1, SAS Institute Inc., Cary, NC, USA) except for experiment 3. The regression analyses were carried out with Sigmaplot software (Ver. 12.5, Systat Software Inc., San Jose, CA, USA). Prior to ANOVA, the percentage data were arcsine-square-root transformed; the data of tuber number and, tiller number and tuber density were square-root transformed; and the data of shoot to root ratio were log-transformed [63]. In experiment 3, the difference of tuber sprouting percentage, sprouts per quadrat and dry weight per sprout among quadrats were analyzed by Kruskal-Wallis non-parametric rank test and Dunn’s post-hoc comparison test (IBM SPSS Statistics V25, IBM Corp., Armonk, NY, USA).

## 5. Conclusions

In the present study, *BPr* exhibited less aggressive competitiveness than *CR* and did not affect the *CR* tuber proliferation in the pots. Different from the results of the pot experiment, both the *BPr* residues and soils exhibited phytotoxicity and reduced the *CR* reproduction in the field. The difference between the results of the pot and field experiments demonstrated that the pot volume might restrict the growth of *BPr* and reduce its effects of allelopathy and competitiveness. However, by combining the results of pot and field experiments, the present study revealed that *BPr* should have the potential for controlling *CR* through its allelopathy in the field.

## Figures and Tables

**Table 1:** Shoot, root and total dry weight per plant of *C. rotundus (CR*) growing in pot treated with the residue of *B. pilosa* var. *radiata* (*BPr*) at four different application rates.

Application Rate, Residue of *BPr* (kg m^−2^)	3 Plants Pot^−1^	6 Plants Pot^−1^	9 plants Pot^−1^
	Shoot dry weight per plant (mg)		
0	451.11 ± 9.09 ^Aa^ (100)	253.89 ± 17.01 ^Ba^ (100)	194.44 ± 15.76 ^Ca^ (100)
0.1	325.00 ± 9.08 ^Aab^ (72)	230.00 ± 14.24 ^Bab^ (91)	193.33 ± 25.00 ^Ba^ (99)
0.2	305.56 ± 84.71 ^Aab^ (68)	217.78 ± 20.21 ^Aab^ (86)	209.26 ± 3.29 ^Aa^ (108)
0.3	291.11 ± 53.48 ^Ab^ (65)	192.78 ± 16.84 ^ABb^ (76)	192.78 ± 7.61 ^Ba^ (99)
	Root dry weight per plant (mg)		
0	512.22 ± 59.92 ^Aa^ (100)	330.00 ± 6.94 ^Ba^ (100)	314.44 ± 22.33 ^Ba^ (100)
0.1	261.67 ± 14.56 ^Ab^ (51)	305.56 ± 28.81 ^Aab^ (93)	313.33 ± 45.03 ^Aa^ (100)
0.2	243.33 ± 85.05 ^Ab^ (48)	255.00 ± 26.03 ^Abc^ (77)	269.26 ± 9.86 ^Aa^ (86)
0.3	224.44 ± 42.92 ^Ab^ (44)	218.33 ± 15.28 ^Ac^ (66)	265.83 ± 18.93 ^Aa^ (85)
	Total dry weight per plant (mg)		
0	963.33 ± 67.41 ^Aa^ (100)	583.89 ± 19.35 ^Ba^ (100)	508.89 ± 37.74 ^Ba^ (100)
0.1	586.67 ± 20.14 ^Ab^ (61)	535.56 ± 39.84 ^Aab^ (92)	506.67 ± 69.15 ^Aa^ (100)
0.2	548.89 ±168.88 ^Ab^ (57)	472.78 ± 22.96 ^Abc^ (81)	478.52 ± 7.52 ^Aa^ (94)
0.3	515.56 ± 83.23 ^Ab^ (54)	411.11 ± 24.95 ^Ac^ (70)	458.61 ± 24.97 ^Aa^ (90)

For each variable, mean ± standard error (*n* = 4) within a row (in superscript capital letter) and within a column (in superscript small letter) followed by the same letter(s) are not significantly different at *p* < 0.05 by LSD test. Data in the parenthesis are percentages of the control (0 kg m^−2^).

**Table 2:** The shoot to root ratio of *CR* growing in pot treated with the residue of *BPr* at four different application rates.

Application Rate, Residue of *BPr* (kg m^−2^)	3 Plants Pot^−1^	6 Plants Pot^−1^	9 Plants Pot^−1^
0	0.90 ± 0.09 ^Ab^	0.77 ± 0.05 ^Aa^	0.62 ± 0.01 ^Bb^
0.1	1.25 ± 0.06 ^Aab^	0.76 ± 0.06 ^Ba^	0.62 ± 0.03 ^Bb^
0.2	1.32 ± 0.10 ^Aa^	0.88 ± 0.14 ^Ba^	0.78 ± 0.04 ^Ba^
0.3	1.36 ± 0.23 ^Aa^	0.89 ± 0.09 ^ABa^	0.73 ± 0.04 ^Ba^

Mean ± standard error (*n* = 4) within a row (in superscript capital letter) and within a column (in superscript small letter) followed by the same letter(s) are not significantly different at *p* < 0.05 by LSD test. Data were log-transformed prior to analysis.

**Table 3:** The tuber and tiller numbers per plant of *CR* growing in pot treated with the residue of *BPr* at four different application rates.

Application Rate, Residue of *BPr* (kg m^−2^)	3 Plants Pot^−1^	6 Plants Pot^−1^	9 Plants Pot^−1^
	Tuber numbers per plant		
0	5.33 ± 0.69 ^Aa^ (100)	3.06 ± 0.20 ^Ba^ (100)	2.86 ± 0.16 ^Ba^ (100)
0.1	3.67 ± 0.14 ^Ab^ (69)	2.94 ± 0.24 ^Ba^ (96)	2.85 ± 0.16 ^Ba^ (100)
0.2	3.11 ± 0.73 ^Ab^ (58)	2.94 ± 0.20 Aa (96)	2.37 ± 0.07 ^Ab^ (83)
0.3	3.78 ± 0.40 ^Aab^ (71)	2.72 ± 0.20 ^Aba^ (89)	2.36 ± 0.08 ^Bb^ (83)
	Tiller numbers per plant		
0	2.78 ± 0.29 ^Aa^ (100)	1.83 ± 0.10 ^Ba^ (100)	1.75 ± 0.05 ^Bab^ (100)
0.1	2.33 ± 0.14 ^Aa^ (84)	1.83 ± 0.00 ^Ba^ (100)	1.56 ± 0.06 ^Bb^ (89)
0.2	2.00 ± 0.33 ^Aa^ (72)	2.17 ± 0.25 ^Aa^ (118)	1.93 ± 0.20 ^Aa^ (110)
0.3	2.44 ± 0.40 ^Aa^ (88)	1.94 ± 0.36 ^Aa^ (106)	1.67 ± 0.08 ^Aab^ (95)

For each variable, mean ± standard error (*n* = 4) within a row (in superscript capital letter) and within a column (in superscript small letter) followed by the same letter(s) are not significantly different at *p* < 0.05 by LSD test. Data in the parenthesis are percentages of the control (0 kg m^−2^).

**Table 4:** Comparison of tuber sprouting percentage, mean sprouts per quadrat and dry weight per sprout of *CR* when sowed in the *BPr* vegetation with (VS) or without (VN) removing the shoots and litter, and in the field (mulched with an opaque plastic sheet, OP) outside the *BPr* vegetation as control.

Treatments	Tuber Sprouting Percentage (%)	Mean Sprouts per Quadrat	Dry Weight per Sprout (mg)
OP	81.00 ± 0.05 ^a^	30.75 ± 2.25 ^a^	9.98 ± 1.76 ^a^
VN	52.00 ± 0.09 ^ab^	18.00 ± 3.03 ^ab^	5.26 ± 0.76 ^ab^
VS	1.00 ± 0.01 ^b^	0.25 ± 0.25 ^b^	1.50 ± 1.50 ^b^

Within each column, mean ± standard error (*n* = 4) followed by the same letter(s) are not significantly different at *p* < 0.05 by Dunn’s nonparametric comparison for post hoc test after a Kruskal-Wallis test.

**Table 5:** In the field severely invaded by *CR*, the influence of *BPr* as cover plants on the tuber density and dry weight per tuber of *CR*. The investigation was conducted on 0 and 69 days after sowing (DAS).

Cover Plants	Tuber Density (tubers dm^−2^)	Dry Weight per Tuber (mg tuber^−1^)
	0 DAS (18 October 2019)	
*BPr*	55.51 ± 5.30 ^a^	98.41 ± 6.68 ^a^
*CR*	60.44 ± 6.90 ^a^	101.12 ± 7.81 ^a^
	69 DAS (26 December 2019)	
*BPr*	92.50 ± 9.59 ^a^	49.51 ± 4.69 ^b^
*CR*	104.91 ± 9.38 ^a^	63.31 ± 4.94 ^a^

Within each column, mean ± standard error (*n* = 12) of different DAS followed by the same letter(s) are not significantly different at *p* < 0.05 by LSD test.
